# Field, Laboratory and Modeling Evidence for Strong Attenuation of a Cr(VI) Plume in a Mudstone Aquifer Due to Matrix Diffusion and Reaction Processes

**DOI:** 10.3390/soilsystems5010018

**Published:** 2021-03-16

**Authors:** Steven Chapman, Beth Parker, Tom Al, Richard Wilkin, Diana Cutt, Katherine Mishkin, Shane Nelson

**Affiliations:** 1 G360 Institute for Groundwater Research, College of Engineering and Physical Sciences, University of Guelph, 50 Stone Road East, Guelph, ON N1G 2W1, Canada; 2 Department of Earth and Environmental Sciences, University of Ottawa, 75 Laurier Ave. East, Ottawa, ON K1N 6N5, Canada; 3 U.S. Environmental Protection Agency, Office of Research and Development (ORD), Center for Environmental Solutions & Emergency Response (CESER), Groundwater Characterization & Remediation Division, Robert S. Kerr Environmental Research Center, 919 Kerr Research Drive, Ada, OK 74820, USA; 4 U.S. Environmental Protection Agency, Region 2, 290 Broadway, New York, NY 10007-1866, USA; 5 U.S. Environmental Protection Agency, Region 3, 1650 Arch Street, Philadelphia, PA 19103, USA

**Keywords:** hexavalent chromium, groundwater plume, matrix diffusion, reaction, attenuation, sedimentary bedrock

## Abstract

This study uses a combination of conventional and high resolution field and laboratory methods to investigate processes causing attenuation of a hexavalent chromium (*Cr*(*VI*)) plume in sedimentary bedrock at a former industrial facility. Groundwater plume *Cr*(*VI*) concentrations decline by more than three orders of magnitude over a 900 m distance down gradient from the site. Internal plume concentrations generally exhibit stable to declining trends due to diffusive and reactive transport in the low permeability matrix as fluxes from the contamination source dissipate due to natural depletion processes and active remediation efforts. The strong attenuation is attributed to diffusion from mobile groundwater in fractures to immobile porewater in the rock matrix, and reactions causing transformation of aqueous *Cr*(*VI*) to low-solubility Cr(III) precipitates, confirmed by high spatial resolution rock matrix contaminant concentrations and comparisons with groundwater concentrations from multi-level sampling within the plume. Field characterization data for the fracture network and matrix properties were used to inform 2-D discrete-fracture matrix (DFM) numerical model simulations that quantify attenuation due to diffusion and reaction processes, which show consistency with field datasets, and provide insights regarding future plume conditions. The combination of field, laboratory and modeling evidence demonstrates effects of matrix diffusion and reaction processes causing strong attenuation of a *Cr*(*VI*) plume in a sedimentary bedrock aquifer. This approach has important implications for characterization of sites with *Cr*(*VI*) contamination for improved site conceptual models and remediation decision-making.

## 1. Introduction

In fractured sedimentary bedrock systems, it is now well-established that diffusion driven transfer of contaminants between mobile groundwater flowing in fractures and the stagnant matrix porewater causes retardation of plume migration and attenuation of contaminant concentrations [1] (pp. 408–413). However, diffusion also impedes remediation efforts due to slow rates of back diffusion from the matrix [2–4]. These effects have mostly been studied for sites contaminated by chlorinated solvent volatile organic chemicals (VOCs) such as tetrachloroethylene (PCE) and trichloroethylene (TCE), in which case matrix storage is enhanced by organic-carbon-dominated sorption. However, without substantial transformation reactions, these chlorinated solvent plumes are considered some of the most challenging sites to remediate and represent long-term liabilities [5,6]. It has been shown that biotic and/or abiotic degradation occurs, albeit slowly, in the rock matrix [7–12] with potential to attenuate concentrations in the matrix and consequently decrease the magnitude and timeframe of back diffusion fluxes. While matrix diffusion effects have been studied at sites contaminated with chlorinated VOCs [13–20] and inorganic contaminants like nitrate [21–23] and chloride [24,25] to our knowledge, these processes have not been examined in detail at bedrock sites contaminated with redox-sensitive heavy-metal contaminants such as chromium. Chromium is a common industrial contaminant globally, ranking 6th of the 25 most frequently detected groundwater contaminants at hazardous waste sites in the U.S. and 2nd (behind lead) for inorganic contaminants [26]. In addition to being released at sites involving metal plating, leather tanning and wood preservation, chromium is also naturally occurring in soils and bedrock in many areas, most typically as insoluble Cr(III) forms, but can undergo oxidation to its more mobile *Cr*(*VI*) form through natural or anthropogenic processes [27,28]. As a consequence, *Cr*(*VI*) occurs frequently in groundwater and surface waters throughout the U.S. [29,30] and worldwide.

**Figure 1.**
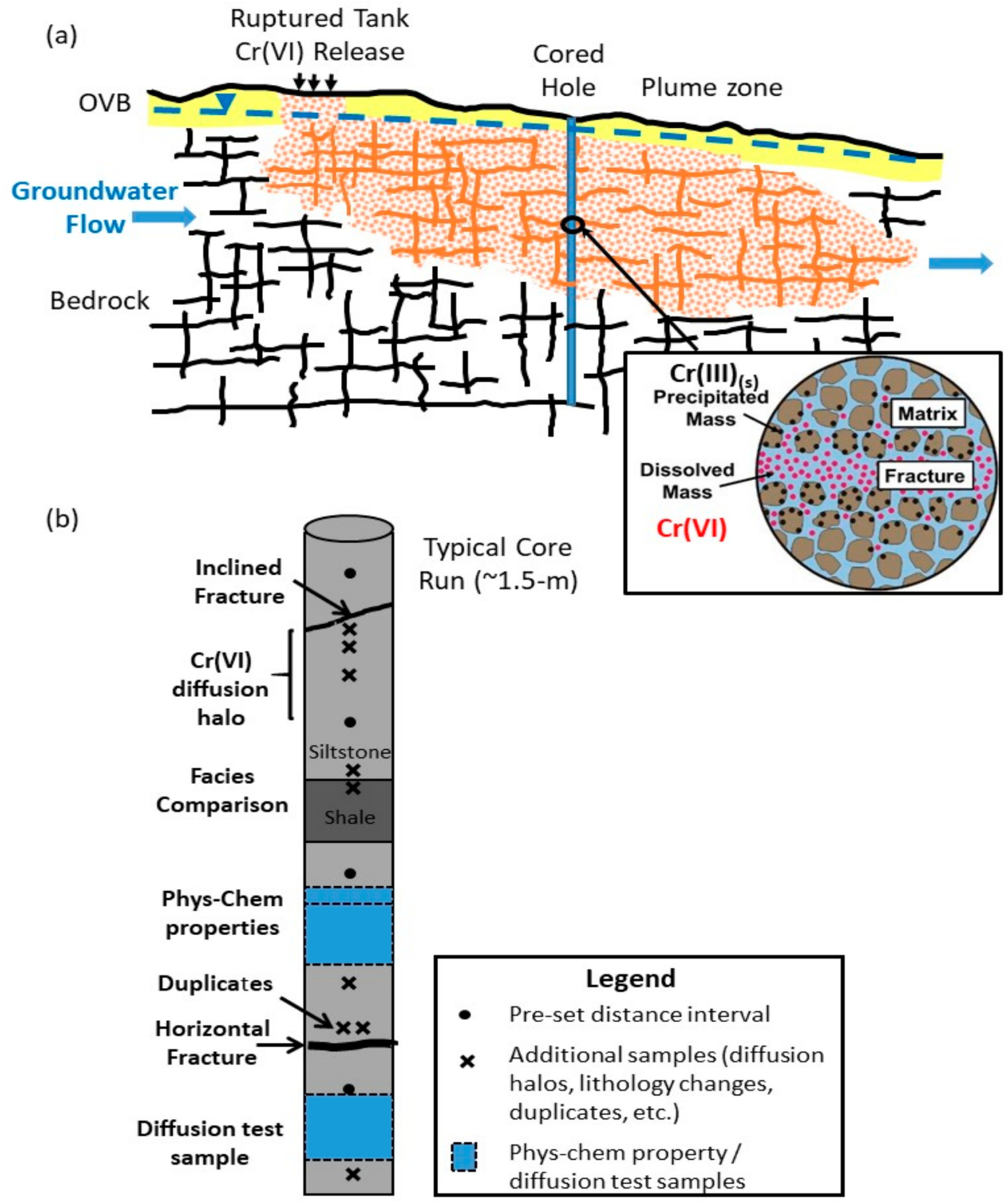
(**a**) Conceptual model for *Cr*(*VI*) plume development and attenuation in fractured bedrock due to matrix diffusion and reaction processes with a cored hole for high resolution investigation; and (**b**) conceptual approach for high resolution core sampling (adapted from Parker et al. 2012).

The approach of selecting one or a few strategic locations within a plume (Figure 1) using high-resolution methods, after advanced stages of site characterization using conventional methods, has been termed the ‘golden spike’ approach. The complement of high-resolution data has been presented as a framework referred to as the Discrete Fracture Network—Matrix (DFN-M) Approach [16]. The value has been to inform key processes influencing matrix—fracture interactions, such as the role of diffusion and reaction conditions within the low permeability matrix blocks between fractures. This approach has been demonstrated at chlorinated solvent contaminated sites in sedimentary bedrock systems [13,14,18–20,31] but not for inorganic contaminants addressed in this study. The DFN-M approach involves a complement of conventional and novel methods applied to characterize both the fracture network and matrix conditions and contaminant behavior/interactions between these environs, including matrix diffusion and reaction processes at a scale commensurate with observed variability. Ultimately the goal is to characterize the fracture network and matrix parameters to allow process-based transport modeling to quantify the matrix diffusion and reaction processes controlling the current plume distribution, and to allow forward-modeling of future conditions and rates of change by natural attenuation and/or with engineered remediation.

**Figure 2.**
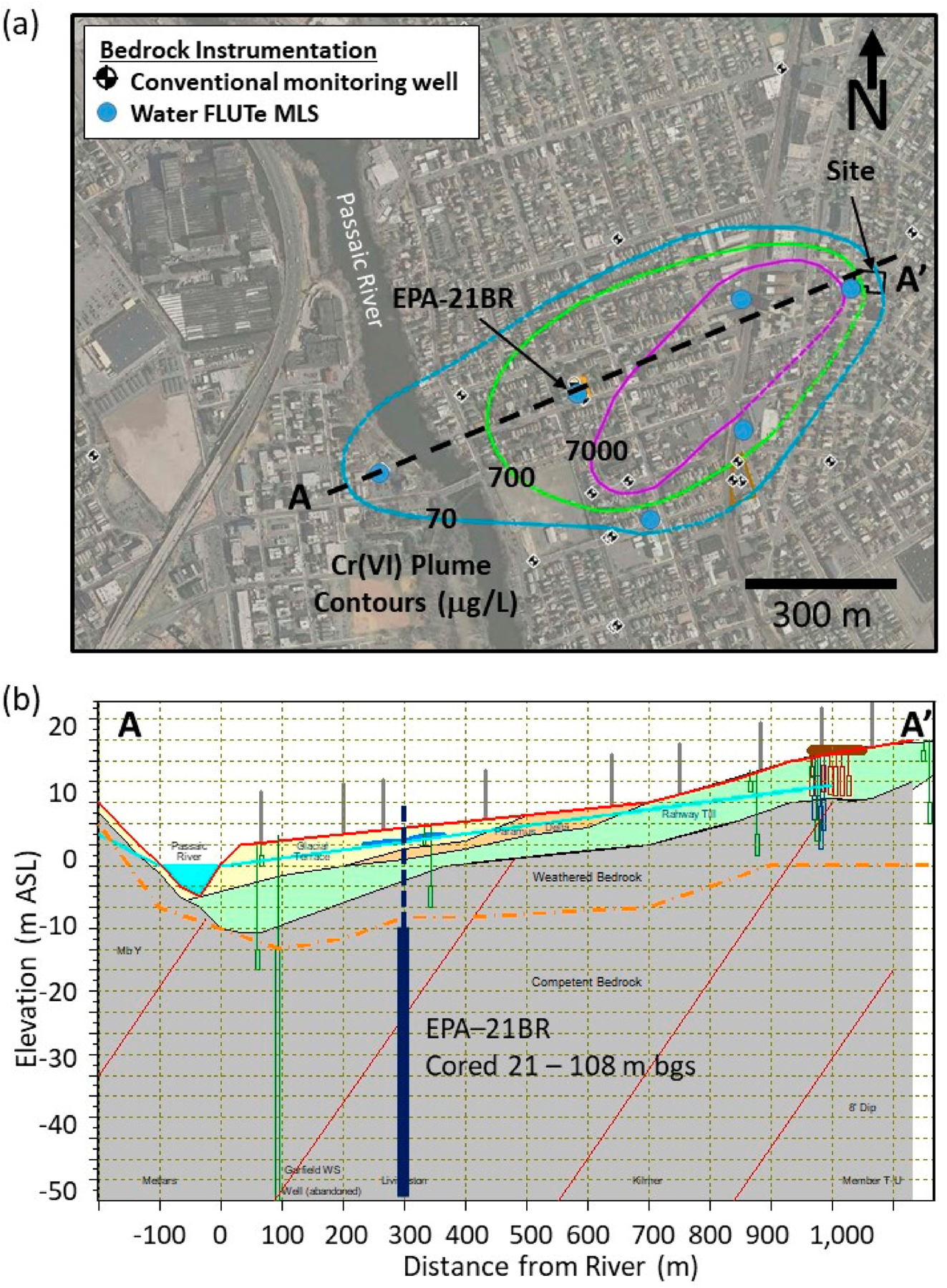
(**a**) Plan map showing the location of the site/release area, bedrock monitoring instrumentation and *Cr*(*VI*) groundwater plume contours in bedrock from 2014 sampling of conventional and multilevel wells (adapted from CH2M Hill, 2015a); and (**b**) cross-section from the site to the river showing general stratigraphy (overburden glacial deposits: green = till, orange = delta, yellow = terrace sediments, weathered and competent bedrock) and site wells. Red lines show the regional bedrock dip of ~8° and dashed orange line shows the approximate interface between weathered and competent bedrock (provided by P. Lacombe, USGS [37]). Location of the cored hole (EPA-21BR) drilled as part of this study is also shown in plan and cross-section.

This study of a *Cr*(*VI*) plume extends the DFN-M approach to *Cr*(*VI*) contamination in a mudstone bedrock aquifer. It utilizes a strategically positioned cored hole (EPA-21BR; Figure 2) with multiple, high resolution vertical profiles that provides the Cr-distribution in bedrock and complementary information about the variability of flow in fractures and matrix properties that affect contaminant distributions. The primary goals of the study were to investigate the bedrock plume extent and degree of attenuation along the flowpath, maximum plume depth and *Cr*(*VI*) mass distribution including evidence for diffusion into the rock matrix by high resolution rock core sampling, and to assess whether evidence exists for redox reactions that reduce *Cr*(*VI*) to Cr(III). Such reduction reactions immobilize Cr and consequently are expected to both enhance attenuation due to matrix diffusion and reduce potential for back diffusion as concentrations in fractures decline as a consequence of reduced source inputs due to natural depletion processes and/or engineered remediation. Laboratory analytical methods were developed for *Cr*(*VI*) extraction from bedrock samples from the study site, providing low porewater method detection limits (MDLs) and minimizing interferences [32]. Further method development also provided the ability to quantify Cr(III) oxyhydroxides in the rock matrix that formed as reaction products from *Cr*(*VI*) reduction providing evidence of enhanced matrix storage and precipitation reactions causing additional attenuation effects [33]. The borehole was subjected to other testing including geophysical logging and hydrophysical testing under sealed conditions using a blank FLUTe™ liner (www.flut.com/blank-liner; accessed on 10 November 2020) recreating natural gradient conditions [34] as well as forced gradient hydraulic testing including transmissivity profiling during liner installation [35] and discrete interval packer testing. These methods provide insights into the position and frequency of hydraulically active fractures and allowed estimation of hydraulic fracture apertures. Subsequently, the borehole was instrumented with a 7-port Water FLUTe™ multilevel system (MLS) [36] (www.flut.com/water-flute; accessed on 10 November 2020) for hydraulic head monitoring and temporal groundwater sampling.

This paper builds on the previous regulatory and method development studies by integrating data from the rock matrix and fracture network to consolidate the evidence for and processes controlling strong plume attenuation. Evaluations included: (1) groundwater *Cr*(*VI*) concentrations in conventional wells and MLS with distance from the site; (2) matrix porewater *Cr*(*VI*) distributions with spacing of samples informed by lithology and varying distances from fractures; (3) comparison of the high-resolution porewater data with groundwater data from MLS ports over the same intervals, (4) comparison of *Cr*(*VI*) concentrations in the porewater with solid Cr(III) concentrations in the rock matrix to demonstrate *Cr*(*VI*) reduction to immobile precipitates; and (5) temporal groundwater *Cr*(*VI*) data from conventional wells and MLS to provide evidence of plume stability. Site data were used to inform a Discrete Fracture—Matrix (DFM) numerical model [38] that simulates matrix diffusion and reaction processes within a statistically generated 2D fracture network, providing quantitative insights on plume behavior. Together the field datasets and simulation results show quantitatively the effects of matrix diffusion and reaction processes that cause strong attenuation of the *Cr*(*VI*) plume with good comparison between simulated and field site contaminant profiles. The simulations also provide important insights on expectations for future plume behavior and for assessment of remedial options.

## 2. Site Description

This study involves a hexavalent chromium (*Cr*(*VI*)) plume in a shallow, sedimentary bedrock aquifer resulting from release(s) from a former electroplating facility in the City of Garfield, New Jersey (Figure 2a). Surficial soils underlying the site are comprised of reworked fills underlain by unstratified glacial deposits comprised of sands, silty sands and gravels with trace silt and clay with thickness ranging from ~5 to 25 m. The overburden generally increases in thickness from the east near the site to the west near the Passaic River (Figure 2b). Below the unconsolidated materials is weathered transitional bedrock grading to competent bedrock of the Passaic Formation, comprised of thinly interbedded reddish-brown sandstones, siltstones and mudstones [37,39]. The water table generally occurs within about 6 m of ground surface within the unconsolidated overburden. Groundwater flow in overburden is generally from the east to west with an average hydraulic gradient of 0.015 and apparently discharging to the Passaic River [40,41]. The potentiometric surface in shallow weathered bedrock follows similar trends as in the overburden. Deeper in bedrock, vertical hydraulic gradients indicate mixed downward and upward flow zones, becoming more upward closer to the Passaic River indicating bedrock groundwater may also discharge to the river [40,41]. At the site, a large documented release of over 2500 kg of *Cr*(*VI*) occurred from a ruptured storage tank in 1983 with possibility of earlier undocumented releases during the period of site operations from the 1930ʹs until facility decommissioning began in 2009. Investigations by the New Jersey Department of Environmental Protection (NJDEP) and the U.S. Environmental Protection Agency (U.S. EPA) and their consultants have been ongoing since the discovery of *Cr*(*VI*) contamination in 1993 and the site was listed under the Superfund program in 2011. These regulatory investigations have included plume delineation in overburden and bedrock with a network of conventional and multilevel wells, borehole geophysical and hydrophysical logging, packer tests, and pilot scale source area remediation trials [40–43]. These investigations have delineated a groundwater *Cr*(*VI*) plume migrating down gradient of the site in overburden and in the underlying sedimentary bedrock aquifer to a maximum distance of ~900 m prior to reaching the Passaic river but with maximum contamination depths poorly defined. Detections of *Cr*(*VI*) also occur in bedrock beyond the river (Figure 2a) indicating it is not a well-defined discharge boundary, presumably owing to flow path complexity in the fractured bedrock system. Uncertainty in contaminant mass distributions between the rapid advection within the fracture network and storage in the low permeability matrix, and how these processes including reactions interact to affect plume fluxes and migration rates, motivated this work.

## 3. Approach and Methods

Investigations at EPA-21BR (Figure 2) included: (1) collection of continuous core using HQ-wireline diamond bit rotary core drilling with a triple-tube core system that better preserves the in situ fracture distribution; (2) core logging for lithology, fractures and other features informing high resolution rock core sampling at fracture surfaces and varying distances into the rock matrix away from fractures (illustrated conceptually in Figure 1b); and borehole testing involving (3) use of inflatable packers to isolate discrete intervals for groundwater sampling and hydraulic testing during drilling [40,44]; (4) geophysical logging of the open borehole including fluid temperature/conductivity, caliper, natural gamma, resistivity and acoustic televiewer (ATV) imaging; (5) FLUTe transmissivity profiling during liner installation [35,45] (www.flut.com/transmissivity-profiling; accessed on 10 November 2020); and (6) Active Line Source (ALS) temperature profiling [34,46,47] in the lined borehole providing insights into the position, frequency and relative flow rates of hydraulically active fractures under forced and natural gradient conditions. A blank FLUTe liner was installed in the borehole after drilling for this testing and to seal the borehole to minimize open-hole cross-connection effects. Following this borehole testing, the borehole was instrumented with a seven port Water FLUTe™ MLS [36] (www.flut.com/water-flute; accessed on 10 November 2020) for depth-discrete sampling and hydraulic head measurements. Some of these methods including packer sampling/hydraulic testing, geophysical logging, transmissivity profiling and Water FLUTe™ MLS installation were also applied at other site boreholes as part of the regulatory investigations.

Groundwater sampling of conventional wells and MLS was conducted during two or three sampling events from 2011–2014 by EPA consultants, including the EPA-21BR MLS. These events utilized standard practices with analyses of field parameters including pH, dissolved O_2_ (DO), oxidation-reduction potential (ORP), temperature and electrical conductivity (EC) and collection of samples for *Cr*(*VI*) and a variety of other geochemical parameters and metals with analyses by commercial laboratories [40,42]. This study included a fourth more comprehensive sampling event of the EPA-21BR MLS in 2016. Purging trials were conducted to evaluate key parameter changes while purging four of the ports through 5–7 cycles (i.e., strokes, with approximately 3 L removed per stroke) using a nitrogen tank with the Water FLUTe™ gas drive sampling system integrated into the MLS. Field parameters (pH, DO, ORP, EC) were measured during each cycle using a YSI-556MPS multiparameter probe along with field measurements of *Cr*(*VI*) (HACH ChromaVer3 colorimetric method) and alkalinity (HACH colorimetric test kit). Significant changes in parameters were observed mostly during the initial two purge cycles with stabilization occurring thereafter, confirming three or more purge cycles were sufficient to provide samples representative of formation groundwater conditions, consistent with FLUTe recommendations (www.flut.com/water-flute-procedures; accessed on 10 November 2020).

The rock core recovered from borehole EPA-21BR was subsampled in detail to allow for high-resolution analysis of *Cr*(*VI*) in the matrix porewater at varying distances from observed fractures in the core logs. The core samples for Cr-distribution (lengths from 3 to 6 cm) were broken out from the HQ-core (approximately 6.3 cm diameter) using a hammer and chisel and preserved by wrapping in foil and then double vacuum-sealed in the field in Mil-Spec film-foil bags to prevent contact with the atmosphere, and shipped on ice to the laboratory. A total of 400 rock core samples for Cr-distributions were collected from the 87.3 m cored interval from 21.0–108.3 m bgs, representing an average sample spacing of 0.22 m. An additional 42 intact samples were also retained from the cores for physical-chemical properties and diffusion tests (sample lengths from 15 to 30 cm) wrapped in foil, parafilm and then sealed in Mil-spec film-foil sleeves to preserve in situ subsurface redox conditions. In the laboratory selected samples were analyzed for Cr-distributions with emphasis on samples overlapping with intervals of the borehole subsequently monitored with the Water FLUTe MLS ports. This was done to reduce the total number of analyses to a manageable level and allow comparisons with groundwater *Cr*(*VI*) concentrations and provide insight into matrix diffusion processes. The MLS ports (3.05 m length) provided groundwater samples expected to represent blended mobile *Cr*(*VI*) concentrations in fractures that intersect these port intervals. In contrast, the rock core sampling provides depth-discrete porewater concentration data in the rock matrix between and adjacent to fractures. Initially a total of 100 samples were analyzed, with average sample spacing of 0.19 to 0.25 m within the plume overlapping with MLS ports 1–5 (<75 mbgs) and 0.19 to 0.38 m below the plume overlapping with MLS ports 6–7 (> 75 mbgs). Subsequently, an additional 46 samples were analyzed to fill in gaps between ports within the Cr contaminated zone.

Laboratory methods were developed for *Cr*(*VI*) extraction/analysis from rock core samples to provide a sufficiently low porewater detection limit (PWDL; 45 µg/L) [32]. This value is lower than the applicable standards with U.S. EPA and NJDEP maximum contaminant level (MCL) of 100 µg/L for total Cr. Matrix porewater concentrations were estimated by transforming the lab-derived total concentrations (*C*_*t*_) using:

(1) $$C_{w}=\frac{C_{t}\rho_{b}-dry}{\phi_{m}}$$

where *C*_*w*_ is the estimated equivalent porewater concentration (µg/L porewater), *C*_*t*_ is the total concentration as a mass of *Cr*(*VI*) per mass of dry crushed rock (µg/g dry crushed rock), *ϕ*_*m*_ is the rock matrix porosity (−) and *ρ*_*b-dry*_ is the matrix dry bulk density (g/cm^3^). A factor of 1000 is also needed to convert from cm^3^ to L. The latter two parameters were measured on a representative subset of samples using gravimetric methods [48]. Subsequently, methods for determining the abundance of Cr(III) precipitates in the matrix were developed [33] which allows quantitation of a mass balance for aqueous (*Cr*(*VI*)) and precipitated (Cr(III)) chromium in the rock matrix.

The high resolution field datasets including rock matrix parameters and fracture network conditions were used to inform and constrain DFM model simulations to quantify matrix diffusion and reaction processes on plume attenuation. The detailed field hydraulic head profiles and rock core contaminant distribution at EPA-21BR are compared with model output as well as the overall degree of plume attenuation along the flowpath based on groundwater monitoring data from conventional and multilevel wells. Details on the DFM model setup and approach is presented later.

## 4. Results

### 4.1. Cr(VI) Plume Status and Trends from Groundwater Sampling

The general groundwater flow direction in bedrock is to the southwest towards the river, which appears to be a regional discharge boundary. The bedrock groundwater monitoring network includes twenty conventional single-interval monitoring wells (most with 3.05 m screen intervals) and six Water FLUTe™ MLS with four to seven monitoring ports (most with lengths of 3.05 m). Groundwater sampling using packers was also conducted at many of the locations during drilling, which helped guide the selection of well screen/multi-level port intervals. Figure 2a shows *Cr*(*VI*) plume contours in bedrock based on sampling of the conventional wells and MLS in 2014, the last plume-wide groundwater sampling episode conducted. Figure 2b shows a cross-section along the plume center-line (i.e., groundwater flow path) with positions of well-screens and EPA-21BR cored interval. Although this is a relatively well-monitored plume, the groundwater *Cr*(*VI*) contours are based on relatively sparse data within the plume, as many of the monitoring wells are located outside of the plume, so the contours provide a fairly simplistic view of the *Cr*(*VI*) distribution. Groundwater data from the monitoring wells and MLS represent *Cr*(*VI*) that is mobile within the fractures that intersect the screen/port intervals blended over these ~3 m intervals. Nevertheless, the data indicate that *Cr*(*VI*) concentrations decline significantly between the source area where the release(s) occurred, and the river, a travel distance of approximately 900 m (Figure 2a).

**Figure 3.**
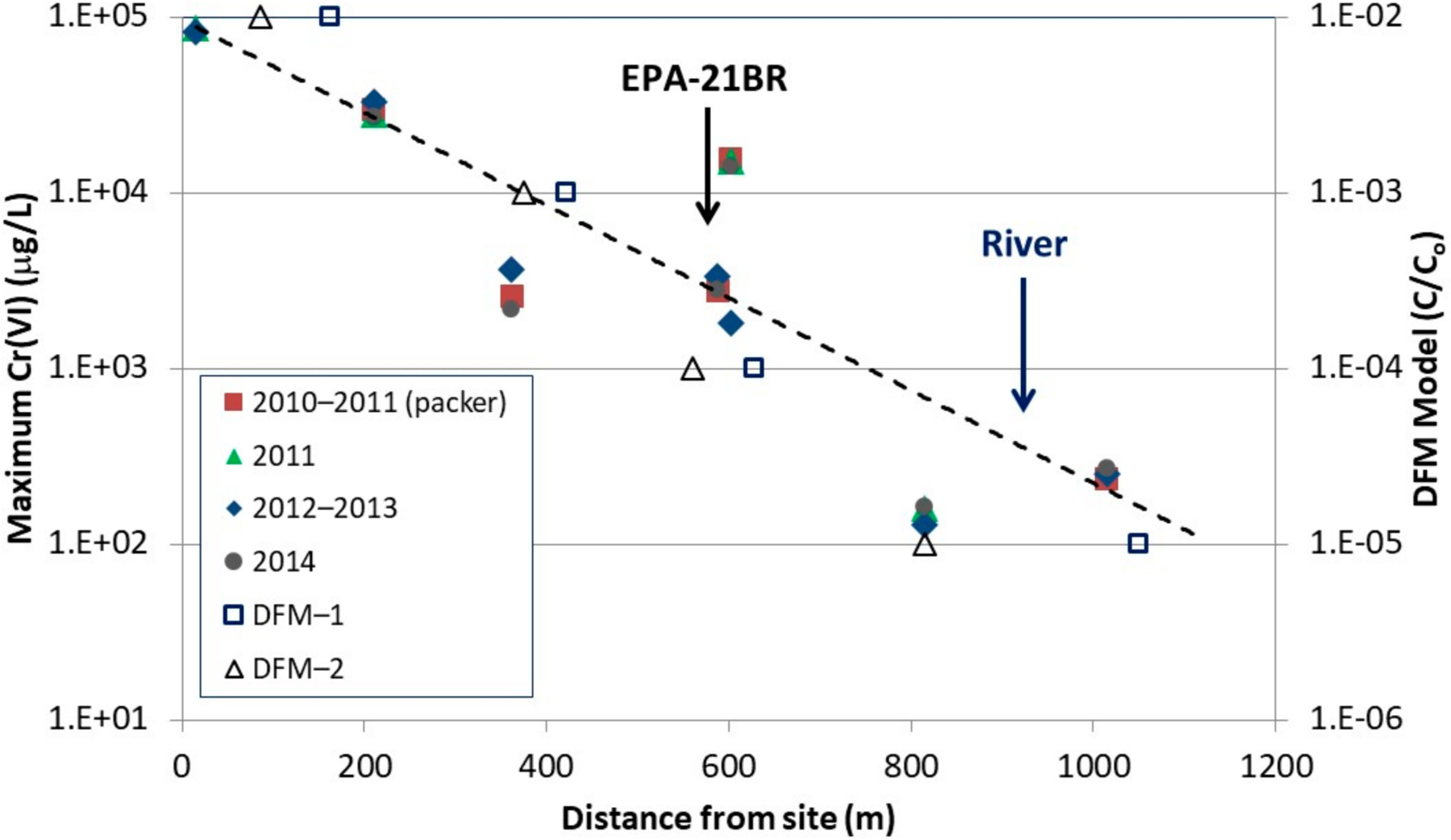
Graph of maximum *Cr*(*VI*) in groundwater along the plume centerline (see Figure 2a) on a logarithmic scale. Data are shown for four sampling events: packer sampling during drilling (2010–2011) and three major groundwater sampling episodes of conventional and multilevel wells in 2011 (Episode 1), 2012–2013 (Episode 2) and 2014 (Episode 3). A logarithmic fit to the maximum *Cr*(*VI*) observed (dashed line) shows a decline by a factor of ~1000 over the ~900 m distance between the site and river. DFM model results (Figure 10) are shown at 30-years representing maximum depth-averaged *Cr*(*VI*) over 3 m vertical intervals, plotted on a relative concentration scale, for comparison with field data (open squares = diffusion only; open triangles = including reaction).

**Figure 4.**
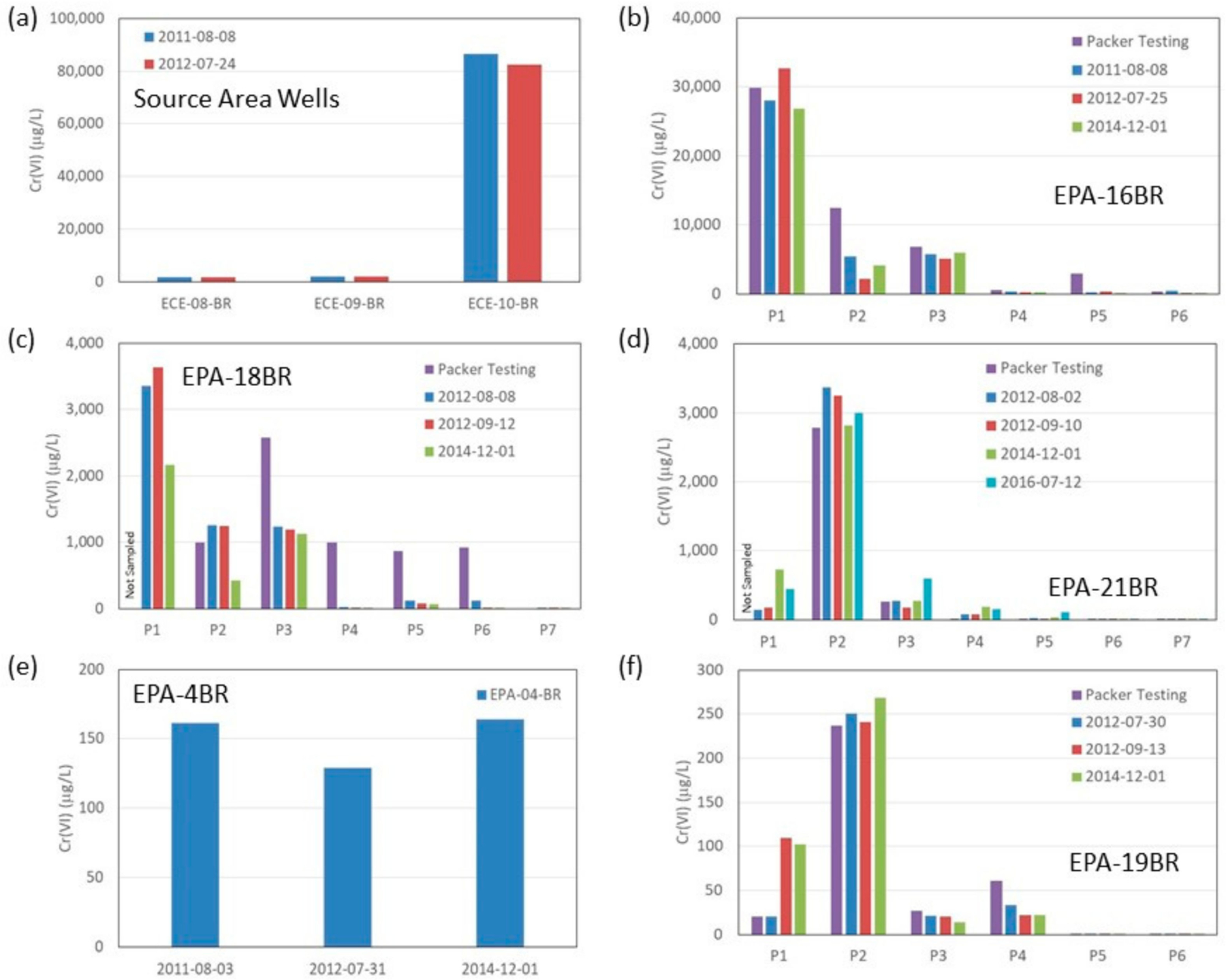
Temporal *Cr*(*VI*) groundwater sampling results for selected conventional wells and multilevel systems along the plume flow path organized by distance from the site (see Figure 2a for locations): (**a**) three source area bedrock wells, (**b**) EPA-16BR MLS, (**c**) EPA-18BR MLS, (**d**) EPA-21BR MLS, (**e**) EPA-4BR conventional well, and (**f**) EPA-19BR MLS. Samples were collected during drilling in 2010–2011 (packer testing) and from major sampling events between 2012–2014 and EPA-21BR sampling event in 2016.

**Figure 5.**
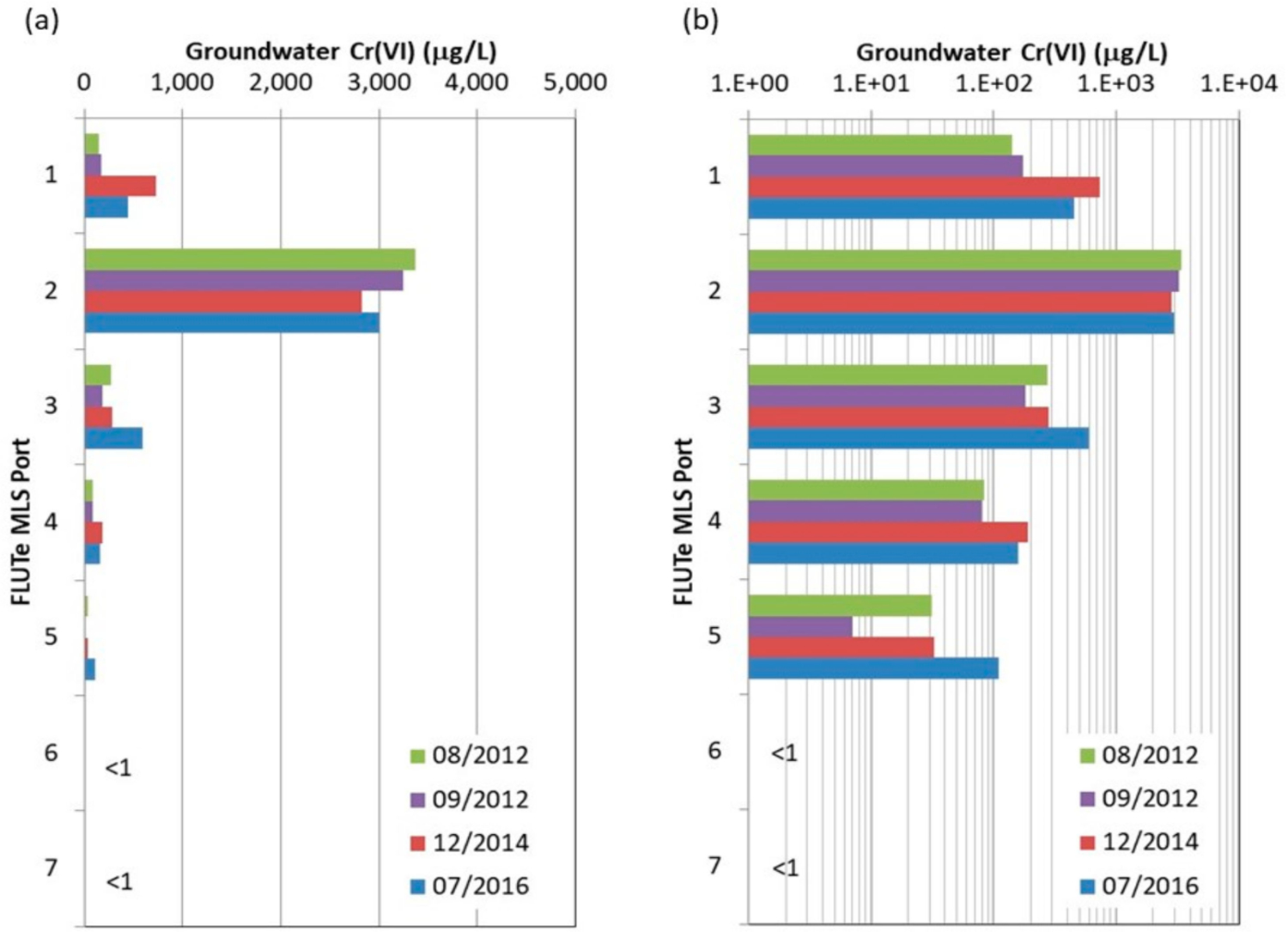
Temporal *Cr*(*VI*) groundwater sampling results from the EPA-21BR Water FLUTe MLS for two sampling events in 2012, one in 2014 and the comprehensive sampling event in 2016, plotted on (**a**) linear and (**b**) logarithmic concentration scales. *Cr*(*VI*) in the two deepest ports (ports #6 and #7) have remained below MDLs for all sampling events.

Trends in groundwater concentrations along the plume flow path are examined in Figure 3. For the MLS, the maximum concentrations observed in any port are plotted. Results from packer groundwater sampling when boreholes were drilled (2010–2011) are included along with three major sampling episodes of the conventional and multilevel wells in 2011 (Episode 1), 2012–2013 (Episode 2) and 2014 (Episode 3). A semi-log fit to the maximum concentrations observed from all sampling events show *Cr*(*VI*) plume concentrations in bedrock decline by over a factor of 1000 between the source and the river. This suggests strong plume attenuation over the 900 m distance given expectations for high groundwater velocities in the fracture porosity alone. *Cr*(*VI*) concentrations in selected conventional and multilevel wells are presented in Figure 4, organized according to sample collection date and distance along the flow path. Although the monitoring duration was relatively short, the data suggest that source zone concentrations in bedrock may be declining (Figure 4a) although this is based only on two sampling events of the three on-site bedrock wells, which were decommissioned during soil removal activities in 2014. This is expected following a single episodic release that occurred decades ago and subsequent remedial activities and natural source depletion processes. Groundwater sampling of on-site overburden wells [42] also shows generally declining trends in *Cr*(*VI*). Similarly, multilevel wells along the flow path (Figure 4b–d) in bedrock also show stable to declining trends within the plume. A conventional well near the river (EPA-4BR; Figure 4e) shows stable *Cr*(*VI*) trends over the three sampling events (range 130–160 µg/L). Stable to declining trends for *Cr*(*VI*) concentrations were also observed in the MLS located west of the river (EPA-19BR; Figure 4f). Migration of *Cr*(*VI*) contamination beyond the river indicates that the river is not a well-defined discharge boundary for bedrock groundwater over the depth interval of contamination. Results from sampling the MLS installed in borehole EPA-21BR (Figure 5) also provide insights on plume stability over the period 2012–2016. The port with the highest *Cr*(*VI*) concentration (port 2) shows a generally stable to slightly declining trend, while the shallower port (port 1) and three deeper ports (ports 3–5) show generally stable to slightly increasing trends. However, these variations are relatively minor and within the margins of error/uncertainty in sampling and analyses, and also are influenced by seasonal variability in the flow system. A longer period of sampling would be required to identify definitive trends. *Cr*(*VI*) was not detected in the two deepest ports (ports 6–7) defining the maximum depth of contamination in bedrock at this location (70 to 85 m bgs). Overall the monitoring data indicate *Cr*(*VI*) concentrations decline sharply with distance down gradient and suggest the bedrock plume is in a stable to declining condition. The plume was further investigated using the DFN-M data sets to inform DFM numerical modeling.

### 4.2. Evaluation of Cr(VI) Matrix Diffusion and Reaction Processes

**Figure 6.**
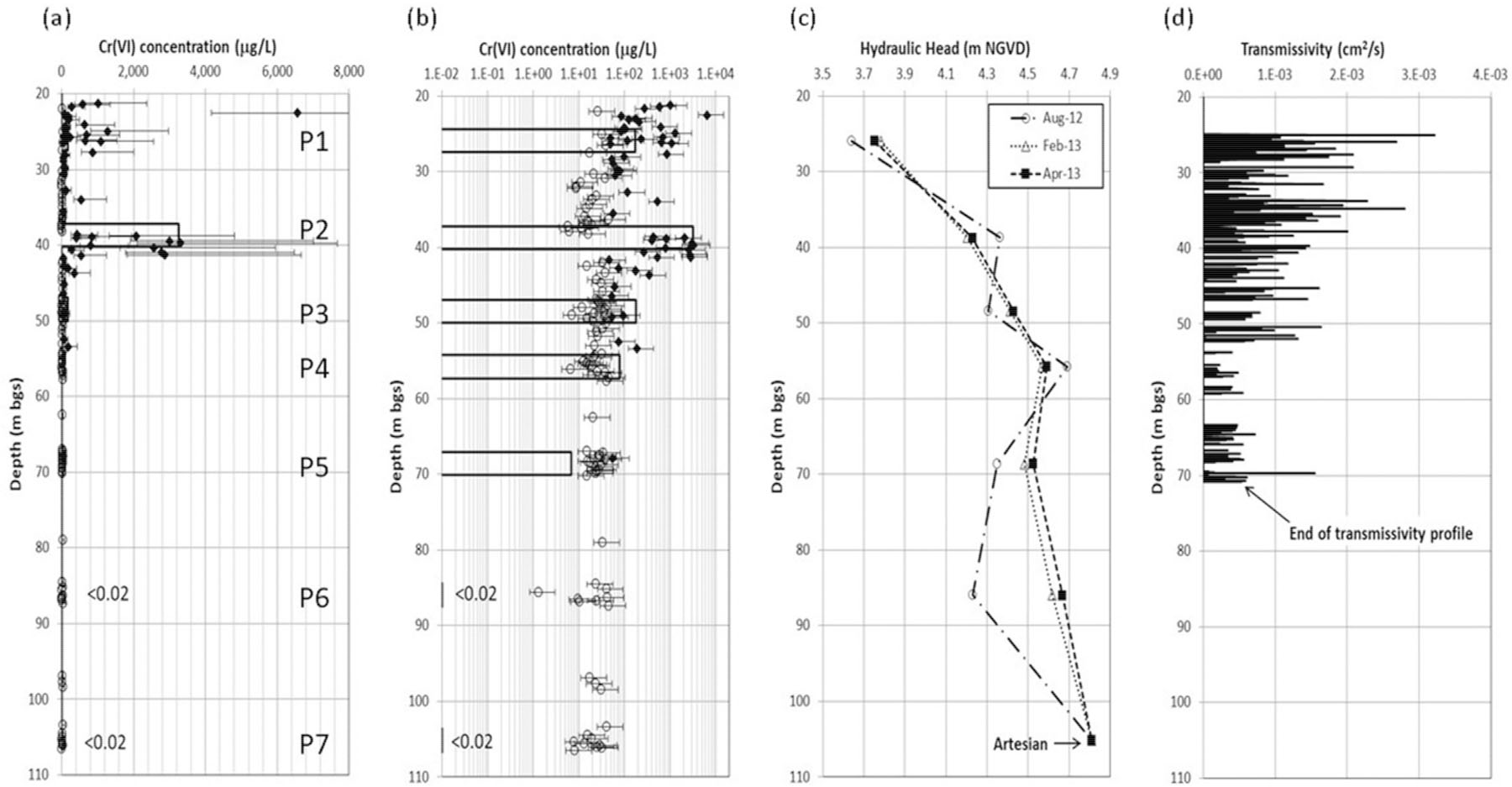
(a) Results of *Cr*(*VI*) rock core analyses for samples collected in April-May 2012 and groundwater *Cr*(*VI*) from MLS sampling (MLS port intervals shown as P1 to P7) in August 2012 plotted on (**a**) linear and (**b**) logarithmic scales. Solid symbols represent positive detections while open symbols represent values that cannot be differentiated from background [32]. Additionally, shown are (**c**) hydraulic head profiles from the MLS, and (**d**) transmissivity profile, which was terminated at ~71 m bgs when the liner descent rate dropped below the testing threshold [35]. Adapted from Chemical Geology, 419, Zhao et al., Determination of hexavalent chromium concentrations in matrix porewater from a contaminated aquifer in fractured sedimentary bedrock, p. 146, Copyright (2015), with permission from Elsevier.

The equivalent porewater *Cr*(*VI*) concentrations are presented in relation to the MLS ports (Figure 6) estimated using mean values for *ϕ*_*m*_ and *ρ*_*b-dry*_ from 36 measurements on core samples (Equation (1)). Error bars represent the range in *C*_*w*_ obtained by applying +/− 1 standard deviation (*σ*) to the mean values (*ϕ*_*m*_ = 0.102, σ = 0.028; *ρ*_*b-dry*_ = 2.46 g/cm^3^, *σ* = 0.10 g/cm^3^). Within the interval represented by port 1, porewater *Cr*(*VI*) concentrations are higher than the blended MLS groundwater concentration by an order of magnitude or more. This suggests that historical *Cr*(*VI*) concentrations in groundwater in this interval were likely higher than at present. The subsequent decline in groundwater concentrations in the fractures creates reverse concentration gradient conditions for back-diffusion from the matrix to the fractures. Within the interval represented by port 2, which exhibited the highest *Cr*(*VI*) groundwater concentrations (3000 to 3370 µg/L), equivalent porewater *Cr*(*VI*) concentrations are highly variable, with values that are below the PWDL in the upper half of the interval and relatively high concentrations in the lower half of the interval, at levels similar to or lower than the groundwater concentration. This suggests strong spatial variability internal to the plume and that groundwater from fractures in the lower half of the port interval dominate the concentrations of *Cr*(*VI*) observed in this port. Elevated porewater *Cr*(*VI*) concentrations in the rock matrix away from any identified fractures indicate pervasive matrix diffusion and accumulation of substantial *Cr*(*VI*) mass in the rock matrix. Within the port 3 interval, the groundwater *Cr*(*VI*) concentrations ranged from 182–274 µg/L, while the equivalent porewater concentrations were below the 45 µg/L PWDL except for two samples (50–95 µg/L) located just below the only fracture identified by ATV in this interval. Within the port 4 interval, groundwater *Cr*(*VI*) concentrations ranged from 80–83 µg/L, while all of the equivalent porewater concentrations were below the PWDL. It is likely that the elevated groundwater *Cr*(*VI*) in port 4 is a consequence of cross-connection that occurred following drilling and prior to installation of the MLS [14]. Within the port 5 interval, groundwater *Cr*(*VI*) concentrations were low, ranging from 7–31 µg/L, and equivalent porewater concentrations were below the PWDL except for one sample (54 µg/L). Within the bottom-most intervals (ports 6 and 7), groundwater *Cr*(*VI*) concentrations were below the MDL (<0.10 µg/L) and all equivalent porewater concentrations in matrix samples were also below the PWDL, effectively defining the lower extent of the plume between 70–85 m bgs. The decrease in concentrations with depth below port 2 is consistent with the transmissivity profile (Figure 6d) which shows the highest transmissivity nearer the top of rock and decreasing transmissivity with successive depths. It is also consistent with the observed hydraulic head profiles (Figure 6c) showing upward vertical hydraulic gradients, indicating flow conditions that would limit the penetration of the plume to greater depth.

**Figure 7.**
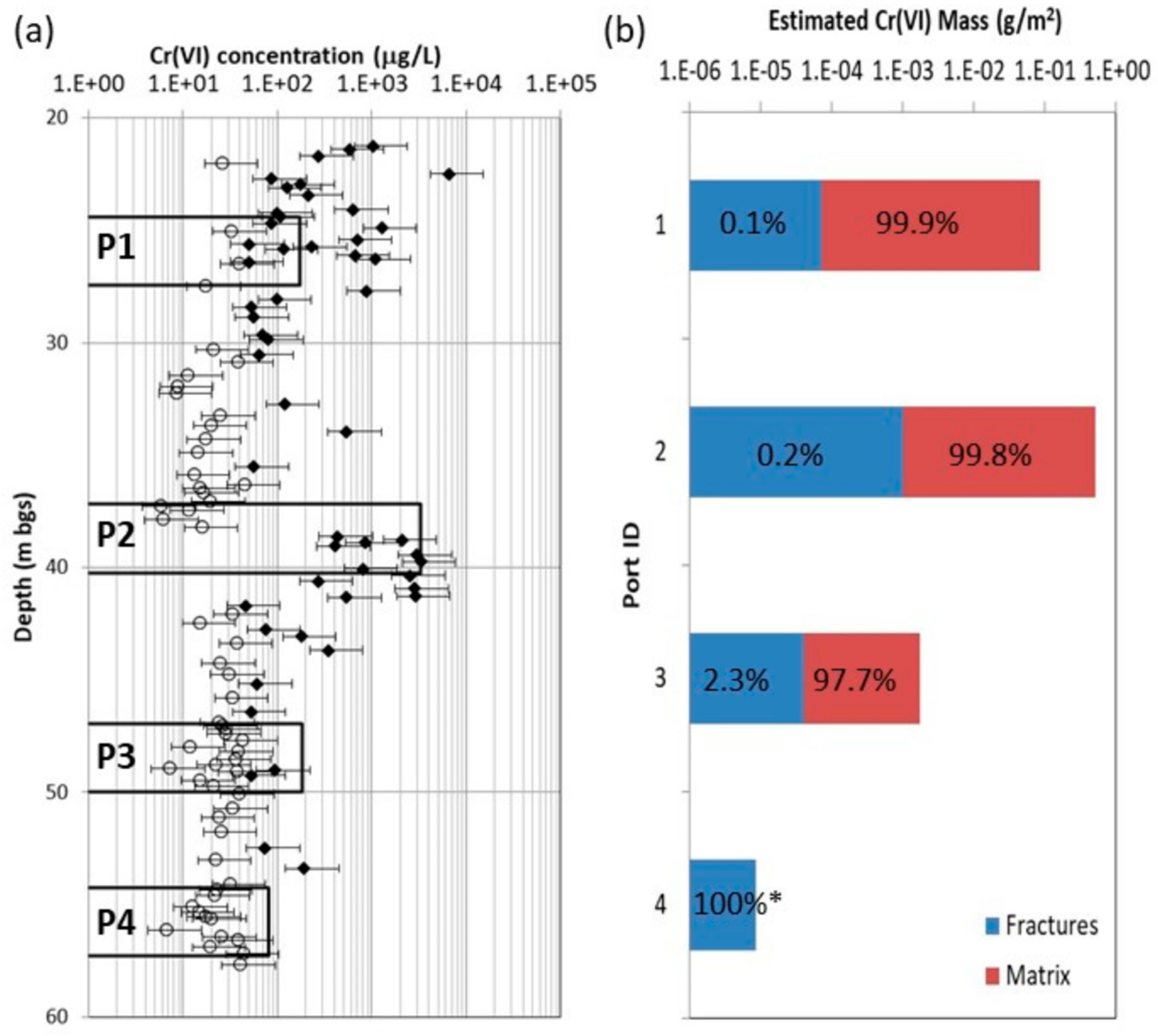
Graphs of (**a**) estimated rock core porewater and groundwater *Cr*(*VI*) from MLS sampling focused on Port #1 to #4 intervals, and (**b**) estimated *Cr*(*VI*) mass within each interval in fractures versus rock matrix porewater with relative proportions indicated. The MLS *Cr*(*VI*) concentrations and relative fracture mass (*) in port #4, and possibly port #3 to a lesser extent, appear to be artifacts of open-hole cross-connection from shallower intervals, given lack of rock core detections in these intervals, which should not be affected by such cross-connection.

Impacts of matrix diffusion processes on contaminant mass distribution between the relatively immobile *Cr*(*VI*) in the rock matrix porewater and more mobile mass in fractures was evaluated with a mass balance calculation in MLS port intervals (Figure 7). Estimates of the mass in fractures (*M*_*f*_) within each port interval were made using:

(2) $$M_{f}=\left[GWCr\left(VI\right)\right]\left[\phi_{f}\right]\left[PL\right]$$

where *GW Cr*(*VI*) is the concentration measured in the MLS port, *PL* is the MLS port length (3.05 m) and *ϕ*_*f*_ is the estimated bulk fracture porosity. The calculation assumes the groundwater *Cr*(*VI*) concentration in each port interval is representative of the mobile groundwater in fractures. Groundwater *Cr*(*VI*) data from the first sampling event in August 2012 were used. Values for *ϕ*_*f*_ for each interval were estimated from the bulk hydraulic conductivity (*K*_*b*_) derived from packer tests and the number of fractures observed in ATV logs within the interval. The *K*_*b*_ values range from 7.1 × 10^−8^ to 8.1 × 10^−7^ m/s for the 7 port intervals. The number of fractures in each interval ranges from 2 to 8. Applying simple calculations for a system of parallel fractures and assuming all fractures within each interval have the same aperture, estimated hydraulic apertures range from 10ʹs to a few 100ʹs of microns and *ϕ*_f_ ranges from 3.3 × 10^−5^ to 1.7 × 10^−4^. These estimates represent “hydraulic” apertures as equivalent parallel plates, whereas real fractures with would likely have larger “physical” aperture regions which may cause underestimation of mass occurrence in fractures.

Estimates of the matrix mass (*M*_*m*_) within each port interval were conducted using:

(3) $$M_{m}=\sum \left[\left[C_{w_{i}}\right]\left[\phi_{m}\right]\left[Z_{i}\right]\right]$$

where the average matrix porosity (*ϕ*_*m*_) and equivalent porewater *Cr*(*VI*) concentrations (*C*_*wi*_) for each rock matrix sample within the interval were applied. The representative vertical interval (*Z*_*i*_) for each matrix sample is the half-distance between adjacent samples above and below it. For these estimates, samples with *Cr*(*VI*) less than the PWDL were assigned a zero value so they do not contribute to the total mass estimate, which may cause underestimation of matrix mass where *Cr*(*VI*) occurs in samples but fall below this level. These estimates also assume negligible *Cr*(*VI*) adsorption within the rock matrix. These estimates also do not include the amount of *Cr*(*VI*) mass that has precipitated within the matrix as Cr(III), discussed below, which would substantially increase the total chromium in the matrix. The estimates for ports 1 and 2, wherein most of the mass resides, indicate that >99.8% of the *Cr*(*VI*) mass occurs in the porewater, demonstrating the significance of diffusion processes toward immobilization and storage of contaminant mass. For ports 3 and 4, the estimates suggest a greater proportion of the total mass occurs in groundwater within fractures. However, the matrix mass estimates are biased low since the PWDL (~45 µg/L) is much higher than the MDL for groundwater analyses (<0.1 µg/L). There were detectable *Cr*(*VI*) concentrations in only two matrix samples from port 3 and none from port 4, with higher *Cr*(*VI*) observed in the MLS groundwater samples. The sparse rock core detections in the port 3 interval is typical near a plume boundary with low level detections nearer fractures and difficult to detect concentrations in the matrix, and the lack of detections in rock core in the port 4 interval provide evidence for the bottom extent of the plume. The *Cr*(VI) detections in MLS port 4 groundwater samples and lack of rock core porewater detections suggests groundwater data was influenced by open borehole cross-connection effects that occurred during drilling, open hole periods, and blank liner and MLS installation episodes, which drives water down the borehole and out deeper fractures. Overall, these mass estimates confirm substantial *Cr*(*VI*) mass in the immobile rock matrix porewater compared to mobile *Cr*(*VI*) in groundwater in fractures.

**Figure 8.**
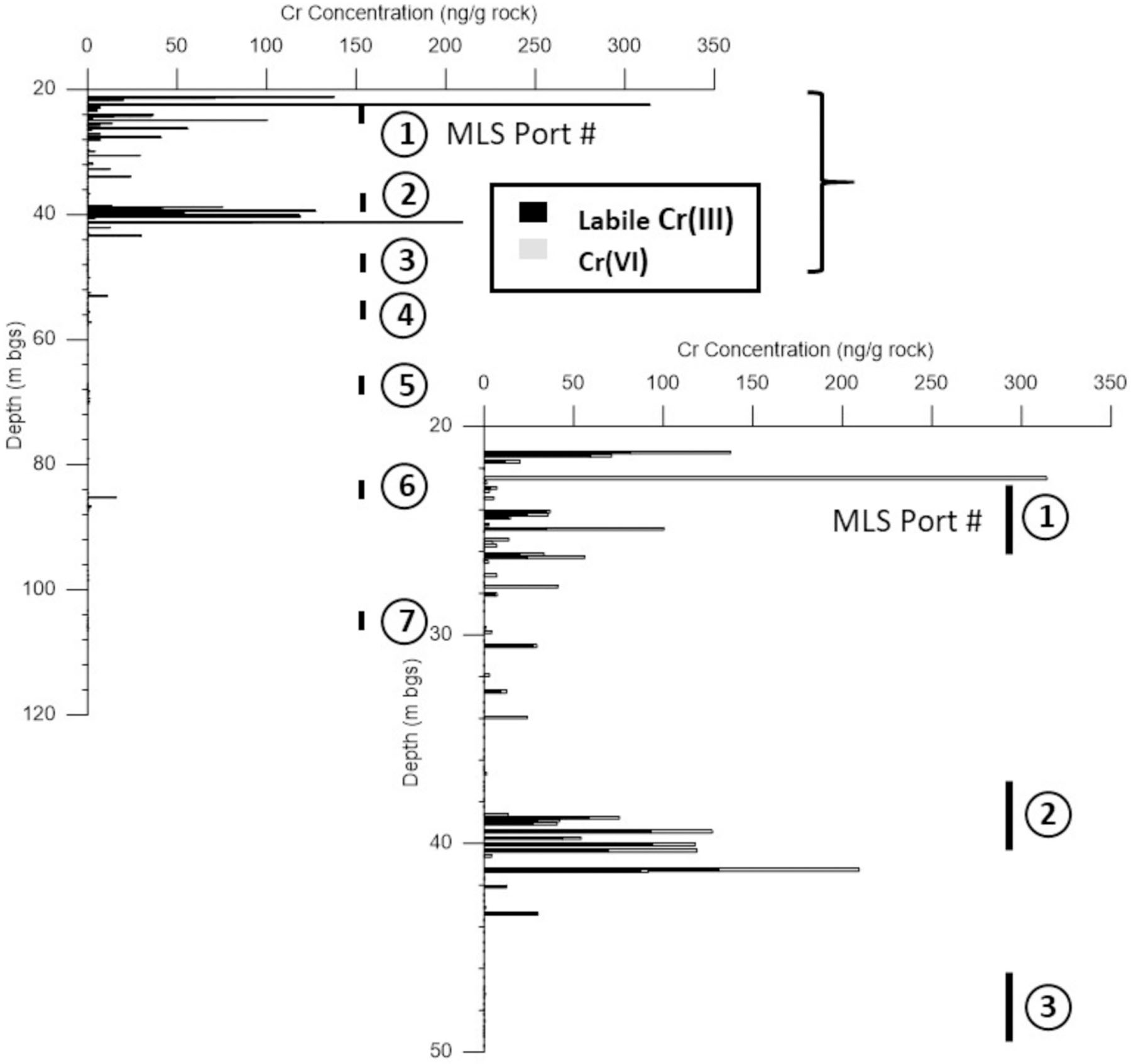
Stacked bars showing background-corrected rock core data for extractable *Cr*(*VI*) and labile Cr(III) for core samples from EPA-21BR, with the grey bars representing concentration of mobile *Cr*(*VI*) and black bars representing labile Cr(III) that formed precipitates in the rock matrix [33]. MLS port intervals are also shown for reference. Adapted from Chemical Geology, 474, Zhao et al., Determination of Cr(III) solids formed by reduction of Cr(VI) in a contaminated fractured bedrock aquifer: Evidence for natural attenuation of Cr(VI), p. 5, Copyright (2017), with permission from Elsevier.

It is expected that *Cr*(*VI*) is susceptible to redox reactions in the rock matrix where reduced Fe(II)-bearing minerals can cause reduction of *Cr*(*VI*) to Cr(III), forming low-solubility Fe(III)-Cr(III) oxyhydroxide precipitates in the pore space [49,50]. Relative concentrations of extractable *Cr*(*VI*) and Cr(III), expressed as a mass of Cr per mass of dry rock (Figure 8), clearly demonstrates that transformation occurs in the rock matrix. In samples with detections of both Cr forms, the ratio of Cr(III) to *Cr*(*VI*) ranged from 0.52 to 22.9 with an average of 4.9, indicating that Cr(III) in many samples exceeded *Cr*(*VI*) concentrations. This was particularly evident in the port 2 interval (see inset in Figure 8) where groundwater *Cr*(*VI*) was highest (Figure 6b). Mineralogical composition assessed using X-ray diffraction (XRD) and scanning electron microscopy with energy dispersive X-ray spectroscopy (SEM-EDS) show that Fe(III)-oxide minerals (e.g., hematite) are abundant but that the pore spaces also contain Fe(II)-bearing silicates (e.g., chlorite, biotite) that are likely responsible for the *Cr*(*VI*) reduction [33]. Removal of *Cr*(*VI*) from matrix porewater via formation of Cr(III) precipitates is expected to enhance matrix diffusion by maintaining higher concentration gradients for inward diffusion from fractures to the matrix. These transformations also reduce potential for *Cr*(*VI*) back-diffusion after the source inputs decline and reduce concentrations in fractures. Long-term stability of the Cr(III) precipitates is of interest, as a change in redox state could result in reversion to *Cr*(*VI*) and release from the matrix. However, it is likely that the stability of the precipitates would increase over time through conversion from amorphous to crystalline structure, such that matrix *Cr*(*VI*) reduction to Cr(III) results in long-term removal of *Cr*(*VI*) from the active groundwater flow system.

### 4.3. DFM Model Evaluation of Plume Attenuation due to Diffusion/Reaction Processes

**Figure 9.**
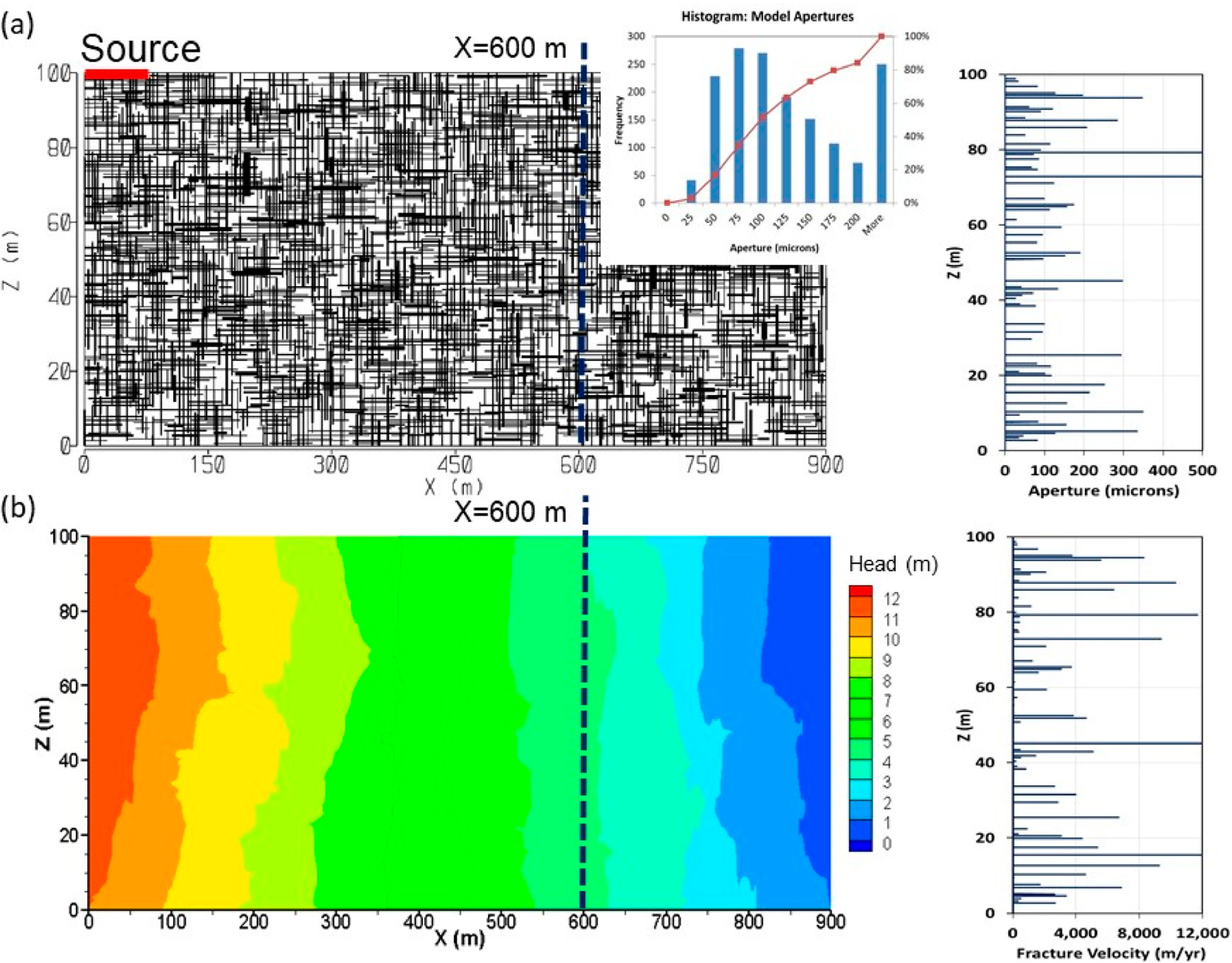
DFM model setup showing (**a**) model domain representing a vertical cross-section along the groundwater flow path and fracture network (inset shows aperture distribution) and example aperture profile at X = 600 m; and (**b**) simulated steady state hydraulic head distribution and example fracture velocity profile at X = 600 m with flow boundary conditions informed by head measurements in conventional and multilevel wells.

Discrete fracture-matrix (DFM) transport simulations were conducted to assess impacts of matrix diffusion and reaction processes on plume behavior within a 2-D model domain that was informed by the DFN-M site characterization. The numerical code FRACTRAN [38] was used, which allows simulation of steady-state groundwater flow and transient solute transport in 2-D in discrete fracture networks compromised of orthogonal fracture sets, generated statistically from a range of fracture apertures, lengths and spacing. The model rigorously simulates advective-dispersive groundwater flow and solute transport in both the fractures and rock matrix, as well as diffusive interactions between them. The code includes the capability to simulate linear sorption on fracture surfaces and in the porous and permeable rock matrix and first-order decay or reaction rates. In these simulations it is assumed that sorption of *Cr*(*VI*) to the fracture surfaces and within the matrix is negligible, although it is recognized that some *Cr*(*VI*) adsorption may occur to mineral surfaces [49,50]. Thus the simulations would be conservative in predicting greater transport rates and lower attenuation than if sorption were included. A generic first-order redox reaction is used as a simplified means to represent *Cr*(*VI*) loss in the matrix due to reduction to Cr(III) and precipitation. The simulations are considered “stylistic” with respect to the fracture network variability, meaning the realization used is not intended to match exact fracture conditions at specific positions in the plume. Rather the goal is to reflect the bulk plume behavior as influenced by flow through the fracture network evaluated by the resultant model domain bulk hydraulic conductivity, assessed by comparison to field tests. The model domain size, hydraulic boundary conditions, fracture network properties (apertures, spacing, length ranges), rock matrix parameters (porosity, diffusion coefficients) and source concentrations as mass inputs were informed by site conditions. Constraints imposed by the model include a 2-D vertical cross-section domain with an orthogonal fracture network, with finite-length parallel-plate fractures with variable apertures and lengths, steady state groundwater flow and simplified source input conditions. Fully 3-D DFM simulations at the plume scale are generally not feasible due to the large number of hydraulically active fractures representative of field conditions, and the discretization requirements within this fracture-matrix system, which makes it computationally impractical to accurately simulate the disparate processes of rapid advection in fractures and diffusion into the matrix across the fracture-matrix interfaces. The 2-D assumption is expected to slightly underestimate attenuation along the centerline of the plume versus fully 3-D simulations, because it does not account for transverse dispersion perpendicular to the 2-D model domain, which also increases the fracture-matrix surface area for matrix diffusion processes [31]. The DFM model domain is 100 m high × 900 m long (Figure 9a) representing a vertical cross-section along the plume center line and groundwater flow path between the source and the river. Grid discretization over the 2-D model domain contains 2157 nodes in the X-direction and 609 nodes in the Z-direction for a total of over 1.3 M nodes and over 210,000 fracture elements. Discretization is refined nearer fractures to accurately simulate matrix diffusion processes. Table 1 summarizes the DFM model parameters. The porosity of the rock matrix was constrained by laboratory measurements, applying a mean matrix porosity (*ϕ*_*m*_) of 10%. An orthogonal fracture network was assigned with fracture spacing, lengths and density for bedding parallel (x-direction) and vertical joints (z-direction) informed by field measurements to the extent possible. Model fracture spacing was informed by the fracture frequency observations from continuous cores and ATV logs, admittedly biased towards lower angle or bedding plane fractures given the vertical borehole orientation used. Based on core logs and ATV logs at EPA-21BR, average fracture frequency was assessed over 3.05 m intervals. Fracture frequencies recorded in core logs ranged from 0 to 3.6 with an average of 1.0 m^−1^ (average fracture spacing of 1.0 m), excluding obvious mechanical breaks. Fracture frequencies measured by ATV ranged from 0 to 2.0, with an average of 0.6 m^−1^ (average fracture spacing of 1.7 m). However, it is expected that both methods likely overestimate the number of hydraulically active fractures significantly contributing to groundwater flow. Core fractures are presumably biased high due to unrecognized mechanical breaks, and since not all identified fractures in cores are expected to represent transmissive fractures. Fractures identified by ATV are subject to uncertainty due to instrument sensitivity and operator interpretation and it is possible smaller fractures may be missed. Additionally, some of the identified fractures may not be transmissive, for example many of the ATV fractures were identified as “minor open” or “partially open”.

Bulk hydraulic conductivity and fracture apertures were informed by hydraulic tests, including transmissivity and K_b_ from packer tests and FLUTe transmissivity profiling along with ALS data indicating hydraulically active fractures. These field derived K_b_ values were used to assess the validity of the statistical fracture networks by comparing with model domain K_b_ derived from flow simulations. As discussed below, several realizations of random fracture networks were generated before arriving at the fracture network most representative of the site conditions used in these simulations. The statistically generated fracture network contains fractures in both x- and z- dimensions that are log-normally distributed with a mean aperture of 1 × 10^−4^ m (100 µm) and variance of log-transformed aperture of 0.5 m^2^. Model apertures exhibit large variability as expected in a natural system consistent with the field derived apertures from packer test data and FLUTe transmissivity profiles [31]. Constant head conditions were applied along the model boundaries providing an overall average horizontal hydraulic gradient of about 1.2% and vertical gradients varying from downward near the contaminated site to upward near the river, generally consistent with field measurements. The source is applied along a 50 m length at the top of the bedrock surface and is finite in time with an assumed initial 20-year duration followed by a stepped decline by 1 OoM each subsequent decade, reducing to zero after 50 years (i.e., C_o_ = 1.0 from 0–20 yr, 0.10 from 20–30 yr, 0.01 from 30–40 yr, 0.001 from 40–50 yr, then 0 thereafter). These conditions are intended to represent the release in 1983 with inputs to bedrock through the overburden, and time for dissipation of the source by natural processes and interim remedial measures, which included early recovery well pumping and pilot scale trials focused on In Situ Reduction (ISR) [43] and with planned overburden and shallow bedrock source area remediation efforts in the near future [52]. Flow simulations were run for several fracture network realizations, tweaking parameters including fracture aperture, density and length ranges until arriving at a fracture network (Figure 9a) judged to be reasonably consistent with the field fracture datasets and target K_b_. Fracture lengths vary from 5–15 m in the z-direction (vertical joints) and 15–75 m in the x-direction (bedding parallel fractures). Using a fracture-stats calculator [51] for the entire domain, average fracture spacing is 1.5 m and 7.7 m for bedding parallel fractures and joints, respectively. For the profile shown in Figure 9a, the average spacing is 1.6 m. The DFM model bedding parallel average fracture spacing of 1.5 m is within the range of estimates based on core (average = 1.0 m), which is expected to be biased high, and ATV logs (average = 1.7 m). Reliable field measurements of joint spacing are not available due to investigations using vertical boreholes. However, the model joint spacing is such that the fracture network is well-connected both horizontally and vertically, supported by mapped joints in sedimentary bedrock systems [53,54].

The simulated steady state hydraulic head distribution with the applied hydraulic boundary conditions is presented in Figure 9b. The overall K_b_ of the model domain is 1.1 × 10^−6^ m/s, whereas values at discrete locations in the domain would vary significantly depending on the local fracture network conditions. The model K_b_ (1.1 × 10^−6^ m/s) is slightly higher than packer test values at EPA-21BR on 8 test intervals (range from 7.1 × 10^−8^ to 8.1 × 10^−7^ m/s, geometric mean of 3.1 × 10^−7^ m/s) and within the range of more broadly distributed packer tests in 6 boreholes on 41 test intervals (range from 3.5 × 10^−8^ to 6.0×10^−5^ m/s, geometric mean of 5.9 × 10^−7^ m/s) [40]. The higher model K_b_ compared to the geometric means from smaller scale packer tests is consistent with expectations for scale dependence of hydraulic conductivity in fractured bedrock aquifers [55,56]. The bulk fracture porosity (ϕ_f_) of the model domain is 9.9 × 10^−5^, which is within the predicted range using estimates from the packer test data at EPA-21BR (3.3 × 10^−5^ to 1.7 × 10^−4^). Simulated groundwater velocities in the fractures are large (see example profile in Figure 9b) providing an expectation of rapid *Cr*(*VI*) transport in the absence of matrix diffusion processes.

**Figure 10.**
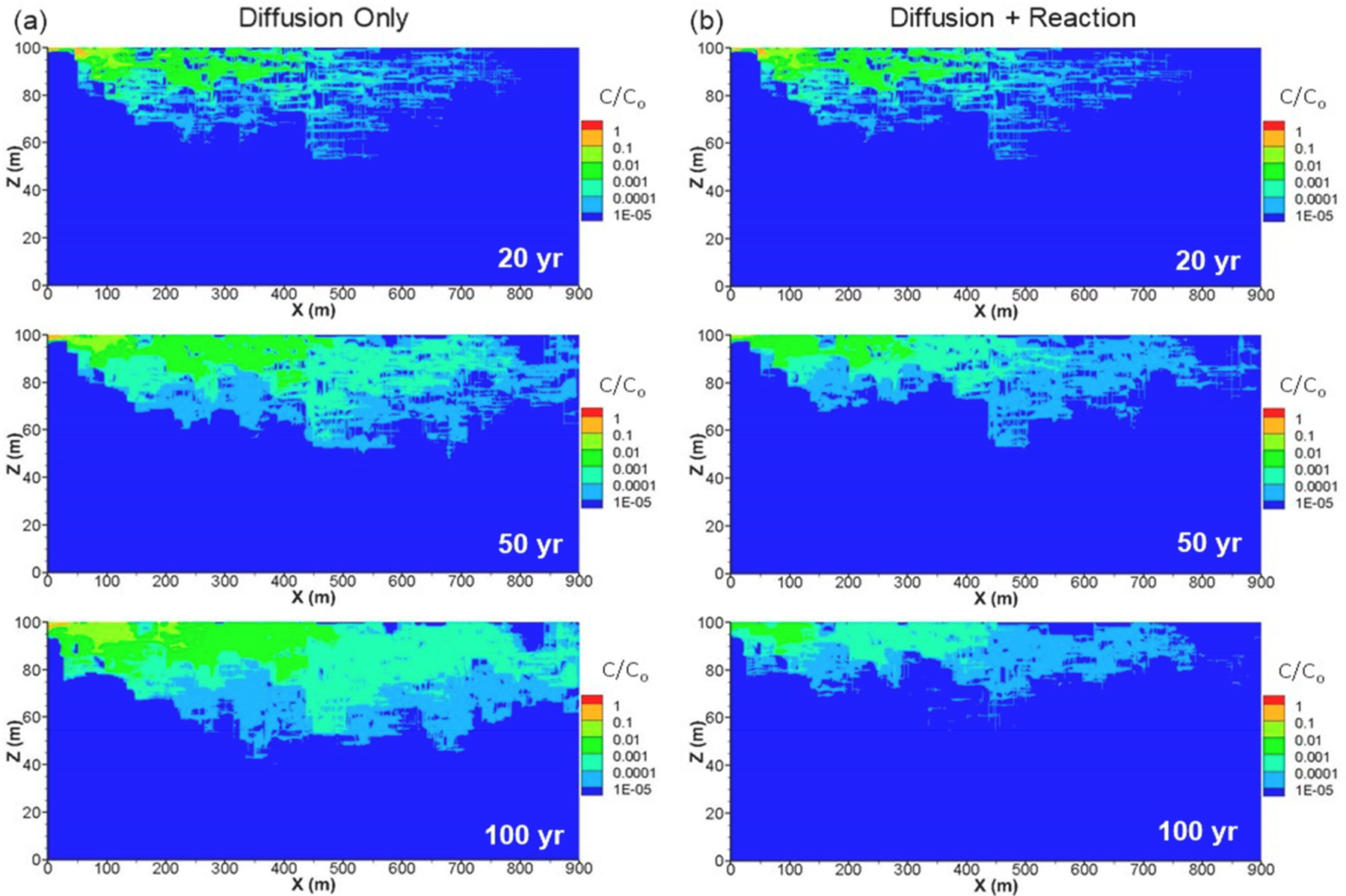
DFM simulated plumes at 20, 50 and 100 years for conditions of (**a**) diffusion only, and (**b**) diffusion and reaction that represents *Cr*(*VI*) loss in the matrix due to precipitation (simplified as first-order decay with t_1/2_ = 20 years). The source is finite with a stepped decline (described in the text) representing the known release in 1983 and assumptions for source dissipation.

Simulated *Cr*(*VI*) distributions at 20, 50 and 100 years without reaction processes are presented in Figure 10a where the plumes are defined by contours of relative concentration (initial source concentration of C_o_ = 1.0). Maximum simulated concentrations are plotted with the maximum field *Cr*(*VI*) concentrations along the flowpath (Figure 3) on a relative concentration scale, representing the same range as the field data, for a 30-year simulation time, consistent with the elapsed time between the *Cr*(*VI*) release (1983) and field groundwater data collection (2010–2014). The DFM model data shown represent maximum depth-averaged concentrations over a 3 m vertical interval, which is consistent with the typical monitoring intervals in wells and MLS ports. The good agreement between field and DFM model results showing similar decreases in *Cr*(*VI*) concentrations along the flow path (Figure 3) highlights the controlling influence of matrix diffusion processes, with large fracture-matrix surface area created by numerous well-connected fractures. Results for simulations that include reactions, approximated as first-order decay with a half-life of 20 years, are shown in Figure 10b. This relatively slow half-life, although still significant over the longer-term when combined with diffusion processes, was selected based on observed *Cr*(*VI*)-Cr(III) ratios at EPA-21BR and elapsed time of 30 years between the 1983 release and the 2012 core sampling. This approach for representing *Cr*(*VI*)→Cr(III) reduction in the matrix is a simplification, but it is still expected to be illustrative of the influence of coupled diffusion and reaction processes on *Cr*(*VI*) concentrations. The simulation that includes reactions (Figure 10b) shows even stronger attenuation because the reaction removes *Cr*(*VI*) from the matrix, causing enhanced transfer of mass into the matrix. These model results suggest the plume might be expected to enter a receding condition as the mass flux from the source declines and is exceeded by the rate of attenuation within the plume. Rates of back diffusion would also be lower with *Cr*(*VI*) removed by redox reactions from the matrix porewater. One key issue then relates to the reactive capacity of minerals in the rock matrix, and whether the Fe(III)-Cr(III) oxyhydroxide precipitates are stable over the long term.

**Figure 11.**
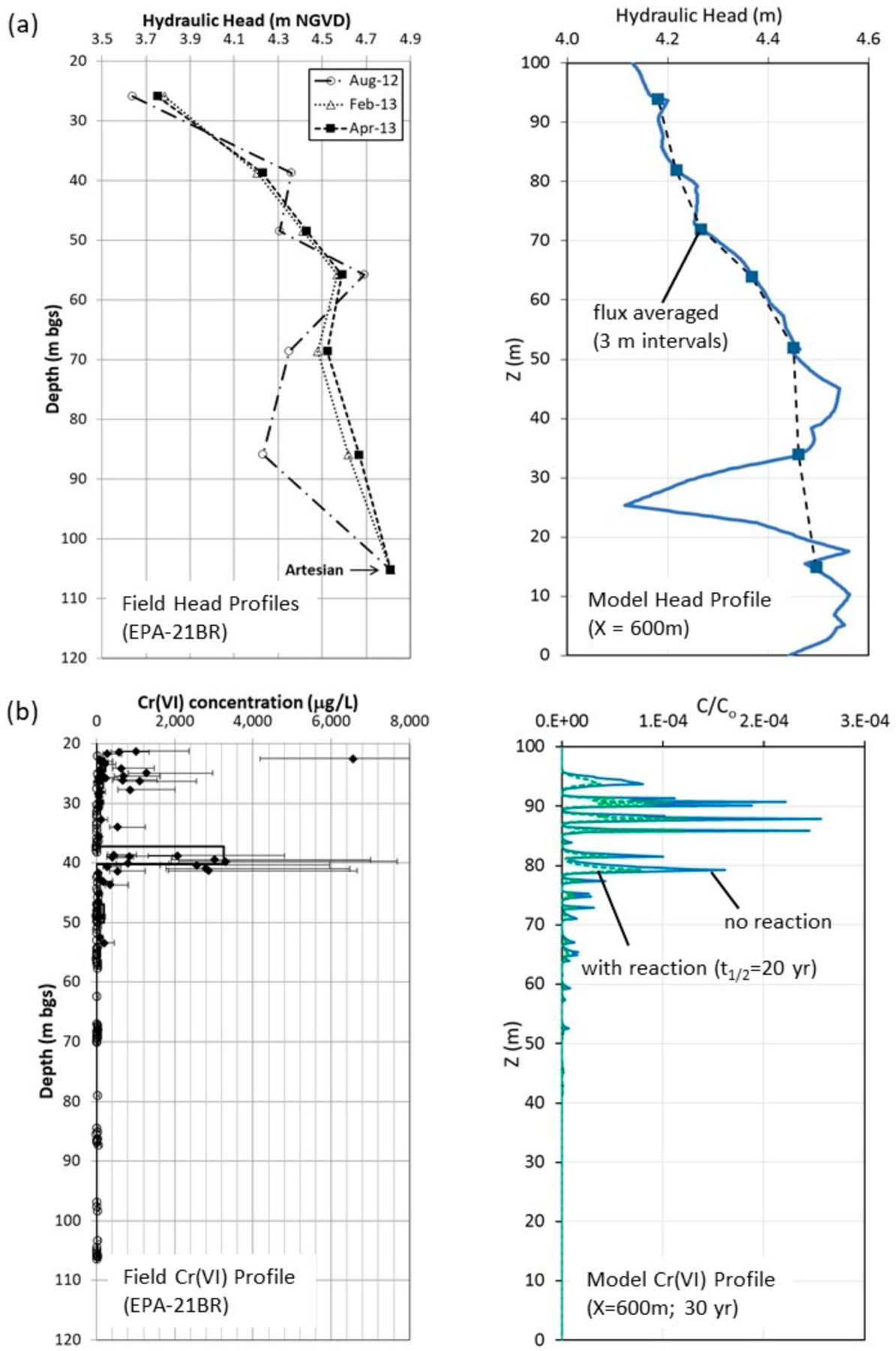
Field versus DFM model comparison of (**a**) hydraulic head profiles and (**b**) *Cr*(*VI*) profiles. The field head profiles in (**a**) were measured in the EPA-21BR MLS and model profiles extracted from DFM flow simulation at X = 600 m (full head profile and flux-averaged values over 3 m intervals at similar relative depths as the field profiles). The field *Cr*(*VI*) profile in (**b**) shows matrix porewater concentrations measured on EPA-21BR core samples and model profiles extracted from DFM simulation at X = 600 m at 30 years for scenarios without (solid line) and with (dashed line) reaction.

Comparisons between field data and model output can be used to judge reasonableness of model outputs for bulk plume behavior, i.e., the plume concentration distributions in the field from monitoring systems. Figure 11a shows a comparison of the field measured hydraulic head profiles in the MLS at EPA-21BR with model profiles extracted from the steady state flow simulation at X = 600 m, representing a similar distance down gradient along the flow path. While the DFM model is not intended to deterministically represent the specific variability of the fracture network, the field and model head profiles show good similarity. The comparison is especially good if flux-averaged head values over 3.0 m intervals (same as MLS port intervals) from the model at similar relative depths as the specific MLS port intervals are applied. Figure 11b shows a comparison between the field *Cr*(*VI*) distribution based on the rock core analyses at EPA-21BR and simulated concentrations from the scenarios with no reaction and with reaction extracted from the model output at X = 600 m at a simulation time of 30 years. The simulated *Cr*(*VI*) distribution shows similarity in style (maximum concentrations in peaks and strong concentration variability over short distances away from discrete fractures) with highest concentrations retained shallow in the flow system.

## 5. Discussion

An ongoing investigation of *Cr*(*VI*) contamination in a sedimentary bedrock aquifer was supplemented with a ‘golden spike’ cored hole providing high-spatial-resolution hydraulic, contaminant-distribution, and geochemical measurements. Conventional monitoring well installations and multilevel wells were useful for broadly delineating plume extent, but did not provide sufficient spatial resolution to understand the transport and reaction processes over decades influencing migration rates, mass flux to receptors and maximum depth of contamination. The monitoring-well data, collected about three decades after the primary release occurred, showed *Cr*(*VI*) concentrations in bedrock groundwater declining by a factor of >1000 over a transport distance of approximately 900 m suggesting strong plume attenuation. Although the monitoring period was relatively short, the groundwater monitoring data also showed that internal plume *Cr*(*VI*) concentrations appear to be stable or even declining.

This study shows how collection of data at high spatial resolution provides mechanistic insights into localized fracture-matrix interactions that control the contaminant mass distributions and create conditions that promote bulk plume attenuation and retardation. The use of rock core for geologic characterization, porewater sampling and determination of the distribution of mobile *Cr*(*VI*) and immobile Cr(III) provide key insights into which fractures conveyed mobile *Cr*(*VI*) and the role of matrix diffusion and redox reactions in attenuating *Cr*(*VI*) concentrations. The rock core investigation included development of new extraction and analytical techniques, which provided ability to quantify the in situ *Cr*(*VI*)-Cr(III) mass balance, avoiding natural interferences and providing low detection limits. The complementary borehole logging methods and hydrophysical testing allowed identification of hydraulically active fractures that informed the position of the multi-depth ports for groundwater sampling. Comparisons of high-resolution porewater concentrations (~0.2 m average spacing) with groundwater data in MLS ports over larger intervals (~3 m) indicated that >99.8% of the *Cr*(*VI*) mass occurs in the rock matrix due to large surface area provided by a dense network of hydraulically active fractures. This is expected for solute plumes in sedimentary rock, but had not previously been demonstrated for a *Cr*(*VI*) plume. Quantitative assessment of the distribution of labile Cr(III) precipitates in the rock matrix [33] demonstrates that mobile *Cr*(*VI*) is effectively transformed to immobile Cr(III), further contributing to plume attenuation and diminishes back-diffusion effects. The mass balance achieved by quantifying both *Cr*(*VI*) and Cr(III) allows for a definition of the boundaries of the contaminated bedrock aquifer. At the location of this detailed investigation, chromium migration is restricted to depths above 45 m bgs, with the Cr mass balance dominated by Cr(III) between 35 and 45 m bgs and by *Cr*(*VI*) at shallower depths above 35 m bgs.

Numerical simulations of chromium transport in a 2-D discrete fracture-matrix network illustrate the influence of solute fluxes into the matrix via diffusion driven by concentration gradients between mobile groundwater in fractures and immobile porewater in the matrix. The numerical framework provides a unique opportunity to assess plume evolution through time, demonstrating the strong influence of matrix diffusion and reactions on *Cr*(*VI*) transport. Simulation results are generally consistent with measured *Cr*(*VI*) concentration distributions in the fractures and the matrix, and reflect the strong attenuation observed along the plume flow path. Simulations that incorporate generic reactions to account for reduction of *Cr*(*VI*) to immobile Cr(III) show enhanced plume attenuation compared to the diffusion-only scenario. Applying simulations to forecast future conditions suggests that plume recession may occur in time scales of 50–100 years after the release occurred. This is consistent with field monitoring data that, although collected over a relatively short time period, suggest that the plume is already in a stable or possibly receding condition. This is a key finding expected to influence the future Record of Decision (ROD) for *Cr*(*VI*) contamination in bedrock (U.S. EPA, 2016). The integration of detailed measurements and modeling allow for a comprehensive appreciation of the processes influencing present and future plume conditions, the time and distance scales over which changes occur, and the risk to receptors. To our knowledge this is the first rigorous study of matrix diffusion and reaction processes demonstrating attenuation of a *Cr*(*VI*) plume in a fractured sedimentary bedrock aquifer. The presence of substantial *Cr*(*VI*) mass in the rock matrix, and conversion from mobile *Cr*(*VI*) to immobile Cr(III), has key implications for assessing monitoring frequency and remediation decision. This includes understanding future plume behavior, and remedial efficacy if there is desire to speed the natural plume attenuation processes.

## 6. Conclusions

This is the first study we are aware of in the published literature where matrix diffusion and reaction processes affecting a *Cr*(*VI*) plume in a sedimentary bedrock aquifer were investigated in detail, at the scale sufficient to measure chromium in the matrix away from multiple, discrete fractures. While groundwater monitoring data collected as part of regulatory investigations showed *Cr*(*VI*) concentration decrease by a factor of over 1,000 along the roughly 900 m plume flowpath, processes causing plume attenuation were not well-understood. In this study the existing site data was supplemented with DFN-M datasets collected from a high resolution cored hole to assess effects of matrix diffusion and reaction processes. This included development of new lab methods for quantification of *Cr*(*VI*) in rock matrix porewater [32] and Cr(III) precipitates in the rock matrix [33] confirming redox reactions, and borehole measurements to assess fracture network/flow conditions. The field and laboratory datasets were used to inform a DFM flow and transport model incorporating key diffusion and reaction processes and their influence on plume behavior.

The primary conclusions from this combined field—laboratory—DFM modeling study are:

- *Cr*(*VI*) mass estimates in mobile groundwater (fractures) versus immobile porewater in the rock matrix, across discrete depth intervals, confirm that the majority of the mass occurs in the rock matrix, demonstrating the importance of matrix diffusion;
- Speciation of chromium into porewater *Cr*(*VI*) and precipitated Cr(III) fractions shows substantial conversion to Cr(III), which is expected to enhance diffusion of *Cr*(*VI*) from fractures into the matrix, and also reduces back diffusion potential of *Cr*(*VI*);
- MLS monitoring showing *Cr*(*VI*) in deeper ports below the plume interval delineated by frequent depth-discrete rock core sampling suggest these deeper occurrences are likely cross-connection artifacts, with the rock matrix sampling data the most reliable indicator of maximum plume depth;
- The DFM model incorporates key controlling processes of matrix diffusion/reaction, providing quantitative insights on process interactions, with output consistent with current conditions, including a detailed rock core *Cr*(*VI*) profile and plume monitoring data demonstrating strong *Cr*(*VI*) attenuation, providing a basis for evaluating future conditions;
- Groundwater monitoring data, although only covering a relatively short time period of a few years, suggests the plume is stable and potentially receding, which is also consistent with the DFM model results;
- The combination of reduced source inputs to bedrock due to natural depletion and overburden remediation and matrix diffusion enhanced by reactions that convert *Cr*(*VI*) to Cr(III) precipitates, is expected to cause slow, continued plume recession into the future;
- The Cr- mass distribution with the majority of the bedrock mass in the rock matrix also has important implications for remedial options and efficacy, if there is a desire to speed these natural attenuation processes.

## Figures and Tables

**Table 1.** Parameters applied in DFM model simulations.

Parameter	Symbol	Units	Range		Value/Mean	Notes
**Rock Matrix Parameters**						
Matrix porosity	ϕ_m_	[−]	−	−	0.10	lab analyses mean
Matrix hydraulic conductivity	K_m_	[m/s]	−	−	1.0 × 10^−8^	literature [1]
Longitudinal dispersivity	α_L_	[m]	−	−	0.10	insensitive (negligible matrix velocities)
Transverse dispersivity	α_T_	[m]	−	−	0.01	
Matrix tortuosity	τ	[−]	−	−	0.10	assumed based on ϕ_m_
Combined Matrix—Contaminant Properties						
Free-solution diffusion coefficient	D_o_	[m^2^/s]	−	−	1.0 × 10^−9^	literature value
Effective diffusion coefficient	D_e_	[m^2^/s]	−	−	1.0 × 10^−10^	calculated D_e_ = D_o_ τ
Matrix retardation factor	R_m_	[−]	−	−	1	assumed (conservative)
Contaminant half-life	t_1/2_	[yr]	−	−	0, 20	assumed (changed in sensitivity analyses)
Fracture Network Properties						
Fracture retardation factor	R_f_	[−]	−	−	1	assumed (conservative)
Fracture dispersivity	α_f_	[m]	−	−	0.10	assumed (insensitive at lower values)
Horizontal fracture density	−	[fracs/m^2^]	−	−	0.02	fitting parameter for target S_H_
Horizontal fracture lengths	L_H_	[m]	15	75	45	fitting parameter for target K_b_
Horizontal fracture spacing	S_H_	[m]	−	−	1.5	P-stats calculator [51]
Horizontal fracture apertures	e_H_	[m]	−	−	1.0 × 10^−4^	field data Informed (packer tests, T-profiling)
Aperture variance (horizontal)	Log σ e_H_	[m^2^]	−	−	0.50	
Vertical fracture density	−	[fracs/m^2^]	−	−	0.02	Assumed
Vertical fracture lengths	L_V_	[m]	5	15	10	Assumed
Vertical fracture spacing	S_V_	[m]	−	−	7.7	P-stats calculator [51]
Vertical fracture apertures	e_V_	[m]	−	−	1.0 × 10^−4^	assumed same as horizontal fractures
Aperture variance (vertical)	Log σ e_V_	[m^2^]	−	−	0.50	
Overall bulk fracture porosity	ϕ_f_	[−]			9.9 × 10^−5^	calculated (model)

## Data Availability

The site data collected by the U.S. EPA and its subcontractors is contained in publicly available reports, which can be found in the ‘Site Documents & Data’ section here: https://cumulis.epa.gov/supercpad/cursites/csitinfo.cfm?id=0206317 (accessed on 20 November 2020). See the reference list [40–43] for links to specific reports. Much of the data generated at the cored location EPA-21BR are provided in Appendix A (Report on Rock Core Sampling and Hexavalent Chromium Analysis at the Garfield Contaminated Groundwater Superfund Site) of the 2014 Remedial Investigation Report available here: https://semspub.epa.gov/work/02/379178.pdf (accessed on 20 November 2020). Additional data presented in this paper related to the Cr(III)–*Cr*(*VI*) rock core assessment are described in Zhao et al. 2017 [33] and can be provided on request from the corresponding author. Data from the associated DFM numerical modeling are not made publicly available due to the difficulty sharing such large model output files in a useful format. Most of the input and boundary conditions for the modeling are provided in the text to ensure others can reproduce the simulations. However additional information including data used to create specific plots and/or model input files can be provided on request from the corresponding author.
